# Eye Movement Variations in Indoor, Outdoor, and Reading Scenarios and Their Implications for Myopia

**DOI:** 10.1155/joph/9954083

**Published:** 2025-11-25

**Authors:** Qi Li, Chao Zhou, Tingting Liu, Yingxiang Han, Zengsheng Chen, Dajiang Wang, Xiaofei Wang

**Affiliations:** ^1^Key Laboratory for Biomechanics and Mechanobiology of Ministry of Education, Beijing Advanced Innovation Center for Biomedical Engineering, School of Biological Science and Medical Engineering, Beihang University, Beijing, China; ^2^Aerospace Center Hospital, Beijing, China; ^3^Senior Department of Ophthalmology, The Third Medical Center of PLA General Hospital, Beijing, China; ^4^State Key Laboratory of Ophthalmology, Optometry and Visual Science, Eye Hospital, School of Ophthalmology and Optometry, Wenzhou Medical University, Wenzhou, China

**Keywords:** eye movements, eye tracking, myopia, reading, virtual reality

## Abstract

**Background and Aims:**

To quantitatively measure eye movement behaviors in indoor, outdoor, and reading scenarios to understand their potential link to myopia.

**Methods:**

Forty-one healthy adult subjects freely viewed indoor and outdoor scenes and performed reading activities using virtual reality (VR). Eye movement data were recorded with the built-in eye tracker of the VR headset (HTC Vive Pro Eye). Gaze and fixation data were calculated and reported for eight regions of the visual field.

**Results:**

Indoor scenes exhibited a more pronounced downward gaze than outdoor environments. Significant differences (*p* < 0.05) in gaze and fixation behaviors were observed between reading and other scenarios. In Region 8 (peripheral inferior visual field), the median (1st quartile and 3rd quartile) number of gaze points was 816 (463, 1175), 1123 (743, 1497), and 1705 (966, 2382) for outdoor, indoor, and reading scenarios, respectively. Similarly, fixation behavior counts were 4 (1, 9), 7 (1, 11), and 39 (22, 54), respectively.

**Conclusion:**

Downward gaze and fixation behaviors are more prevalent in reading and indoor environments. Given that downward eye movements can induce instantaneous axial elongation, our results suggested a potential biomechanical pathway for myopia progression through optic nerve traction and ocular tissue remodeling. This study underscores the need for further research to explore the specific role of eye movement behaviors in the progression of myopia, especially in real-life settings.

## 1. Introduction

The global prevalence of myopia, particularly in East Asia, has become a major public health issue [1, 2]. Beyond impairing vision quality, myopia can progress to pathologic myopia, leading to severe complications such as foveoschisis, retinal detachment, and glaucoma [1]. Despite extensive research, the exact mechanisms underlying myopia remain unclear.

Education and intensive near-work activities, such as reading, are major risk factors for myopia [2]. Consistent evidence has shown that outdoor activities can effectively slow down myopia progression [3, 4]. However, the underlying mechanisms are not fully understood. Current research largely focuses on the role of light exposure [5, 6], but the findings have been mixed, with some [7] supporting the hypothesis and others presenting contradictory evidence [8–10]. Besides light intensity, other factors might be in play in the development and progression of myopia. For example, studies have shown that reading has a greater impact on students' myopia progression than light intensity [11, 12].

Our previous studies [13–16] and others' [17, 18] have shown that the optic nerve traction force in the anteroposterior direction during eye movement could deform the posterior eye globe, potentially leading to growth and remodeling of ocular tissues. Consistent with these findings, adduction and downward gaze have been linked to transient axial length increases, independent of accommodation effects [19–21]. In addition, interventions such as prism lens wear, which reduce convergence eye movement, have been shown to slow myopia progression [22]. These findings suggest that eye movement behavior across various environments and activities may contribute to myopia progression, yet the specific patterns of these movements have not been previously studied. Therefore, the aim of this study was to quantitatively measure eye movement behaviors in indoor and outdoor environments and during reading, using eye tracking technology combined with virtual reality (VR).

## 2. Methods

In this study, three distinct scenarios were used to analyze eye movement characteristics: viewing indoor scenes, viewing outdoor scenes, and reading. Eye movement data were recorded under these scenarios to observe variations in gaze and fixation patterns.

### 2.1. Subject Recruitment and Characteristics

This study involved 41 healthy adult subjects (mean age = 24 years, standard deviation = 3 years, 24 males and 17 females). The recruitment and experimental procedures were conducted at Beihang University from July to December 2020. All subjects had myopia with refractive errors ranging from −1.00 to −6.00 diopters, which were measured using an autorefractor. Each subject wore corrective glasses to achieve optimal visual acuity during the study. Exclusion criteria included the presence of abnormal stereo vision, strabismus, retinal disease, visual field defects, or other eye diseases, as well as restrictions in body movement and balance, difficulties in comprehending or adhering to the instructions provided by the research team, or an inability to engage in the full extent of the experimental protocol.

### 2.2. Ethical Approval and Informed Consent

The study was approved by the Biomedical Ethics Committee of Beihang University (BM20200051, March 30, 2020) and was conducted in accordance with the guidelines of the Declaration of Helsinki. Written informed consent was obtained from all participants.

### 2.3. VR Setup

The VR device used in this study was the HTC VIVE Pro Eye head-mounted display (HMD), featuring a built-in eye tracker provided by Tobii (Tobii AB, Stockholm, Sweden). The eye tracker offers a data output frequency of up to 120 Hz and an accuracy range of 0.5°–1.1°. Raw data of the eye tracker can be exported using the HTC SRanipal software development kit.

To simulate both indoor and outdoor scenes, the DeoVR video player was utilized. 360° panoramic images were projected into the VR's 3D space using equirectangular projection. To simulate reading scenes, the VR Kindle application was utilized. The e-book was displayed in 2D panel mode, allowing the user to adjust the location of the virtual Kindle to achieve a comfortable reading distance.

### 2.4. VR Scene Selection and Categorization

**Figure 1:**
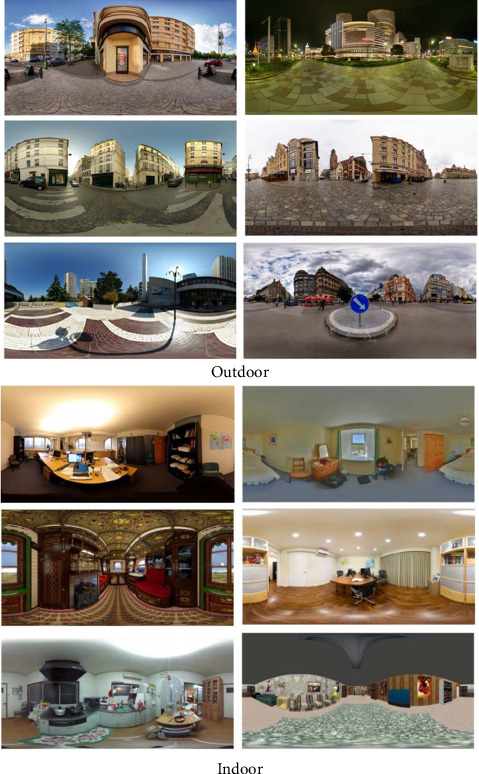
Panoramic images of the indoor and outdoor scenes used in this study. The outdoor scene primarily consists of urban open spaces, and the indoor scenes feature various room spaces.

VR scene images were sourced from a 360° panoramic image database compiled by Rai et al. [23] From this collection, we selected 12 images, depicting indoor spaces (6 images) and urban outdoor spaces (6 images), reflecting our participants' typical environments and the higher myopia risk in urban settings. Rural scenes were excluded to preserve a clear indoor–outdoor contrast and avoid confounding visual statistics. The resolution of the chosen images was greater than 5760 × 2880, facilitating the creation of clear, immersive environments.  Figure 1 displays the 360° panoramic views of the scene images for the indoor and outdoor settings.

### 2.5. Experimental Procedure and Data Recording

Before the experiment, each subject underwent an eye movement calibration. Following this, we launched the DeoVR scene player and data recording applications to initiate the experiment. At the start, subjects were oriented to face the center of the image by following guidance arrows to locate a target at the image's center. They were instructed to close their eyes and rest until a countdown reached zero, at which point they opened their eyes to freely explore the VR environment. During the free exploration, no further instructions were given until the exploration ended.

Subjects viewed each image for 25 s, consistent with the protocol of Rai et al. [23]. Subsequently, they were reoriented to the image's center and closed their eyes for a 10 s rest before transitioning to the next image, compared with Rai's 6 s [23], to enhance participant comfort and reduce potential carryover effects. These durations are sufficient for fully exploring the 360° panoramic image and restoring cognitive resources, drawing on with the protocol of Rai et al. [23] For each subject, the images were presented in a fixed order to ensure that all participants experienced the same sequence, eliminating the variability caused by sequence effects as confounding factors.

After viewing all the scene images, subjects engaged in reading a book in Kindle VR for 180 s until the phrase “experiment ends” appeared in the VR scene. The duration for reading was chosen to reflect typical sustained engagement and to approximate the cumulative exposure time of viewing 360° images.

Subjects were offered breaks after viewing each group of images to prevent symptoms of discomfort such as dizziness or nausea, which can arise from VR incompatibility. If a break was needed, they could remove the HMD and rest. Before resuming, the eye movement calibration step was repeated.

The recorded eye movement data included timestamps, gaze origin and gaze vector, data reliability, pupil size, and eye openness for both eyes. Only the data from the left eye were analyzed, as the data from both eyes were similar.

### 2.6. Data Processing and Eye Movement Parameters' Calculation

The raw data were exported as text files and later converted to .csv files for data processing. The beginning of each viewing session was identified using the eye openness data, as subjects were instructed to close their eyes before starting tasks. Subsequently, a segment of 25 s of data following the beginning was extracted, representing the eye movement data for that specific image.

In the indoor and outdoor scenarios, for each scene image of a single subject, the eye movement behaviors were analyzed. The eye movement parameters were then calculated for each image of each subject. For reading, recorded data were segmented into six consecutive 25 s epochs, which were analyzed to enable direct comparison with the indoor and outdoor.

Eye movement behavior includes two major types: fixation behavior and saccade behavior. Fixation was identified using the dispersion threshold identification (I-DT) algorithm [24]. The dispersion threshold of 1° and duration threshold of 150 ms were used in the algorithm.

### 2.7. Measurements of Gaze and Fixation Behaviors

**Figure 2:**
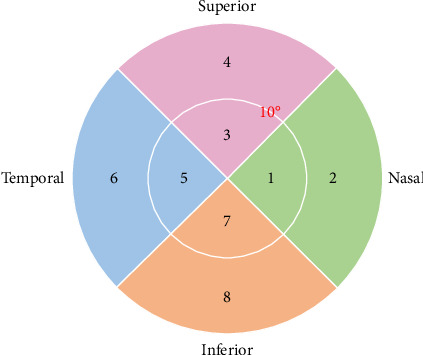
Illustration of the 8 regions of the visual field used in this study. The visual field was divided into four sectors: nasal, superior, temporal, and inferior. Each sector was further divided into two subregions, with a 10° boundary for central vision.

Gaze behavior was represented through gaze points in the visual field. Specifically, gaze points were obtained by projecting the gaze vector onto a spherical surface centered on the eye [25]. These gaze points on the spherical surface were then plotted as a 2D map, similar to a standard human visual field map. The visual field was divided into four sectors: nasal, superior, temporal, and inferior. Each sector was further divided into two subregions, with a 10° central vision boundary line as shown in Figure 2.

The density of gaze points on the visual field was plotted using a probability density map [26, 27]. High-density area indicates where subjects gaze more frequently, as eye position data were recorded at a constant frequency. For each scene within the indoor and outdoor scenarios, gaze points for each subject were analyzed.

The gaze points or regionwise gaze points for each individual subject were calculated separately for all 6 scenes in both indoor and outdoor scenarios. For reading, gaze points were calculated for each subject during six consecutive 25 s periods to align with the six images per scenario in both indoor and outdoor scenarios, ensuring consistent duration and data segments across all conditions.

For fixation behavior, individual fixation locations in the visual field and fixation durations were obtained using the I-DT algorithm. These fixation points were then plotted as a probability density map, similar to the gaze behavior analysis. Subsequently, the number of gaze events at each sector for a specific subject across different scenarios was calculated, mirroring the approach used for gaze behavior analysis.

### 2.8. Statistical Analysis

Statistical analyses were conducted in accordance with established principles in medical statistics and with reference to the Statistical Analyses and Methods in the Published Literature (SAMPL) guidelines [28, 29]. Prespecified comparisons of gaze and fixation behaviors across indoor, outdoor, and reading conditions were assessed using Mann–Whitney *U* tests, with Bonferroni correction applied for multiple comparisons. Exploratory analyses of downward gaze patterns (Region 8) were performed using Mann–Whitney *U* tests with Bonferroni correction. A significance threshold of *p*=0.05 was used for all two-sided tests. Continuous variables are reported as median (IQR), and actual *p* values are presented in tables and figures. All analyses were performed using Python (V3.10) with statsmodels (V0.14.4) and SciPy (V1.10.1).

## 3. Results

### 3.1. Gaze Behavior

**Figure 3:**
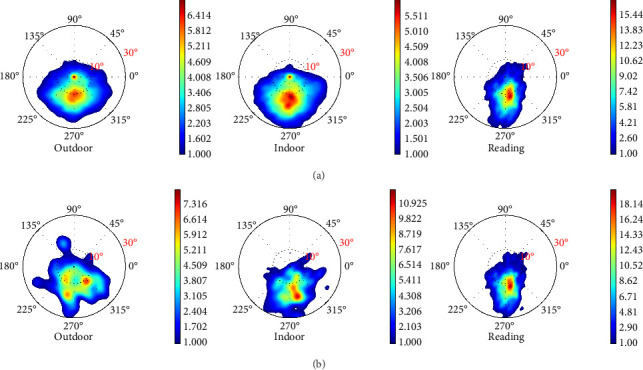
(a) Gaze points probability density map on the visual field in all three scenarios. (b) Fixation points probability density map on the visual field in all three scenarios. Note that the color scale ranges in each subfigure vary to better illustrate the relative distribution within each scenario.

The probability density map in Figure 3(a) illustrates the gaze behavior across three scenarios: viewing indoor scenes, viewing outdoor scenes, and reading. The probability density of gaze points in reading (maximum probability density of around 19) is much higher than that in indoor (maximum probability density of around 6) and outdoor (maximum probability density of around 7). Gaze points were largely concentrated in the inferior regions (Regions 7 and 8) for all three scenarios.

**Figure 4:**
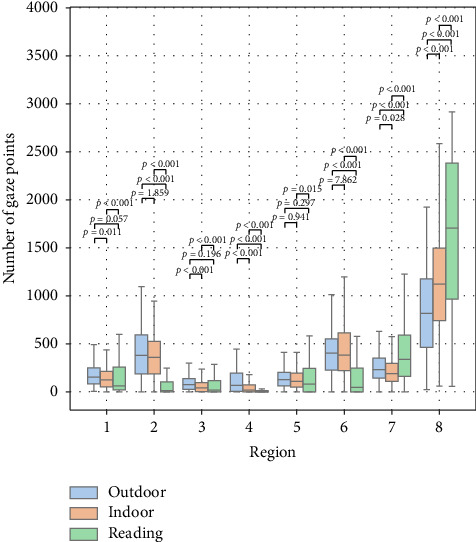
Box plot of the number of gaze points in different regions across all three scenarios.

Furthermore, the number of gaze points in different regions for all subjects in each scenario is shown in Figure 4. There were significant differences between the reading and the other two scenarios. The number of gaze points in Region 8 is also listed in Table 1. The number of gaze points in Region 8 was highest during reading, followed by the indoor scenario, and then the outdoor scenario, suggesting more frequent eye movement toward the inferior direction in reading and indoor scenarios.

The number of gaze points on the nasal and temporal sides (Regions 2 and 6) in reading is obviously less than that in indoor and outdoor. That indicates that the majority of the gaze points were within a relatively narrow region in reading compared to indoor and outdoor scenarios.

### 3.2. Fixation Behavior

**Figure 5:**
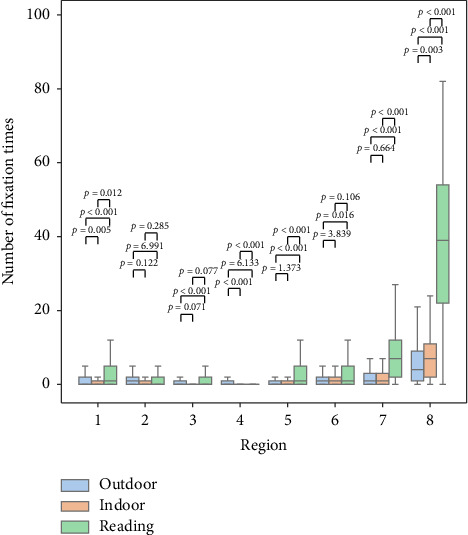
Box plot of the number of fixation times in different regions across all three scenarios.

Figure 3(b) shows the probability density of fixation points.  Figure 5 shows the number of fixation times in different regions for all subjects across the three scenarios. There were significant differences between the reading scenario and the other two scenarios in Regions 1, 5, 7, and 8. The number of fixations in Region 8 is shown in Table 1. There was also a significantly higher number of fixations in the reading and indoor scenario than in the outdoor scenario.

## 4. Discussion

In this study, we found distinct eye movement behaviors across various scenarios, with a notably higher frequency of downward gaze and fixation during reading compared to both indoor and outdoor settings. Indoor environments also exhibited more downward gaze and fixation than outdoor ones. According to previous studies [13–18], downward eye movement has the potential to cause transient axial elongation and could potentially have longitudinal effects.

Our findings highlight a fundamental difference in the eye movement patterns associated with reading, compared to those observed in outdoor and indoor environments. Inferior areas consistently showed more gaze points and fixation behaviors across all scenarios, particularly in reading. Notably, both indoor and reading scenarios showed a higher count of these points compared to outdoor scenarios.

Reading is a well-known risk factor for myopia. Our data showed that reading involves more downward gaze and potentially larger accumulative optic nerve pulling in the anteroposterior direction, while outdoor activity has the least downward eye movement. Our previous study [14] has shown that the traction force of the optic nerve on the eyeball during eye movement is comparable to the force exerted by extraocular muscles and acts in the anteroposterior direction. This traction force can deform the optic nerve head, and the deformation can be larger than that induced by a substantial intraocular pressure elevation [13, 15]. Optic nerve head deformations induced by eye movement have been shown to be correlated with normal tension glaucoma [30]. Mechanical stress is known to influence tissue growth and remodeling, a concept referred to as “stress-modulated growth” [31]. While it is plausible that sustained or repetitive optic nerve traction may contribute to mechanical stimuli relevant to ocular remodeling, its role in axial elongation remains speculative. Currently, there is no direct histological or longitudinal clinical evidence in humans confirming this mechanism. Current evidence supporting this hypothesis is limited and indirect. Finite element modeling has suggested that optic nerve traction may induce posterior globe deformation [14, 16]. Clinical studies have reported transient increases in axial length during downward gaze or near work, implying a possible mechanical component [19, 20, 32]. In animal models, particularly the guinea pig myopia model, early elongation has been observed near the optic nerve insertion rather than the macula [33]. These findings raise the possibility that the optic nerve traction could be a factor related to myopia. This study provides behavioral-level observations that may indirectly support the hypothesis. However, longitudinal studies incorporating direct anatomical or biomechanical measurements are needed to validate this mechanism.

Time spent outdoors is known to be protective against myopia, but the underlying mechanism remains elusive. Previous studies have primarily focused on the role of light exposure during outdoor time [5, 6], with some suggesting that moderately bright light increases retinal dopamine levels, thereby offering some protection against myopia [7]. However, there are also studies that challenged this hypothesis [8, 9], which argued that light may not be the actual mechanism [10]. The spatial frequency characteristics of different scenes may be one of the missing drivers of the form deprivation myopia [34]. Our results suggested that eye movement behaviors differ during outdoor activity compared to indoor and near work, such as reading. It is a new hypothesis that these differences in eye movement behavior may be one factor contributing to the effectiveness of outdoor activities in mitigating myopia progression. Importantly, the mechanical consequences of gaze direction may also play a role and warrant further investigation. Prior biomechanical studies have shown that adduction generates greater optic nerve traction than abduction [15, 35], with strain magnitude influenced by optic nerve tortuosity [36], orbital anatomy, and tissue properties [37]. Although these studies focused on lateral gaze, biometric data suggest that downward gaze may induce even greater transient axial elongation [19]. Nevertheless, the longitudinal impact of both downward gaze and adduction on tissue growth and remodeling remains unclear, and further research is needed to determine their relevance to myopia development.

Previous studies on eye movement behaviors in different scenes have primarily focused on the restorative effects, i.e., the recovery from mental fatigue and stress, and their impact on the physiological and psychological conditions of observers in natural scenes compared to urban and modern architecture [38–41]. These studies focused on visual attention toward targets, not the eyeball. In contrast, this study examines the eyeball's movement behaviors across different scenarios. Furthermore, we used a head-mounted eye tracker to eliminate the effects of head movement, allowing us to measure eye movement behaviors while subjects freely moved their heads and eyes. This approach is different from other methods that restrict head movement to a small range. Moreover, we employed VR technology to conduct experiments. Previous studies have shown that 3D cues are fundamentally different from those in 2D pictures, which only provide intensity, texture, and color cues [42]. The VR technology allowed subjects to observe the scene in a more immersive manner, differing from other eye movement studies that used a laptop screen to display a scene and asked the subject to view it from an aerial perspective, which is far from reality.

In this study, especially in the indoor scenario, subjects were asked to explore freely in the room. However, this is not typical behavior for most people during indoor activities, which usually involve specific tasks. For example, students usually remain indoors for academic activities such as attending classes and studying. At home, they might engage in completing homework, reading, or preparing for exams, along with other indoor leisure and screen time. Therefore, real-life indoor scenarios often involve more near work compared to the tasks in this study, likely resulting in a more pronounced downward gaze due to these activities. However, even for free viewing tasks, subjects still move their eyes more in the downward direction due to the region of interest or object of interest being more concentrated below eye level, showing more downward gaze and fixation behaviors. Further studies that accurately replicate everyday indoor activities will enhance our understanding of eye movement behaviors in indoor spaces.

In this study, we used a fixed task order to standardize exposure and to simplify headset and software handling, thereby reducing interruptions in VR tracking. Although the relatively short total duration makes significant eye fatigue unlikely [43], we cannot exclude potential order effects. Future studies with randomized task orders will help to further elucidate whether such effects play a role.

We used gaze and fixation patterns to quantify eye movement behaviors in this study. Prior studies have indicated that greater eye movement magnitude is associated with increased traction force of the optic nerve [44], thereby exerting more force on the posterior sclera of the eyeball. However, the precise influence of force duration at a specific magnitude remains unclear. It is plausible that brief, instantaneous forces, such as those experienced during saccades, might have a lesser effect on tissue growth and remodeling compared to a prolonged period of sustained force of the same magnitude and cumulative duration during fixation. Elucidating this requires a fundamental understanding of scleral growth and remodeling processes. Further investigations into the sclera's response to varying force magnitudes and durations are warranted to clarify this point.

## 5. Conclusion

This study identified unique patterns of eye movement across different scenarios, with a notably higher frequency of downward gaze and fixation during reading and indoor scenarios compared to outdoor settings. Such persistent downward eye movements may be associated with an increased risk of developing myopia, suggesting a potential link that warrants further investigation.

## Figures and Tables

**Table 1:** Number of gaze points and fixation times in Region 8.

	Scenarios (median (IQR))			Pairwise *p*-values		
	Outdoor	Indoor	Reading	Outdoor vs. indoor	Outdoor vs. reading	Indoor vs. reading
Number of gaze points	816 (463, 1175)	1123 (743, 1497)	1705 (966, 2382)	< 0.001	< 0.001	< 0.001
Number of fixation times	4 (1, 9)	7 (2, 11)	39 (22, 54)	0.003	< 0.001	< 0.001

## Data Availability

All data supporting the findings of this study are included within the article. The raw datasets generated and analyzed during the current study are available from the corresponding author upon reasonable request.
